# Association of cardiovascular-kidney-metabolic index with all-cause mortality during hospitalization in critically ill patients: a retrospective cohort study from MIMIC IV2.2

**DOI:** 10.3389/fcvm.2024.1513212

**Published:** 2024-12-09

**Authors:** Xiaolong Qu, Yuping Liu, Peng Nie, Lei Huang

**Affiliations:** ^1^ Department of Cardiovascular Medicine, Renji Hospital, Shanghai Jiaotong University School of Medicine, Shanghai, China; ^2^ Department of Nutrition, Gongli Hospital of Shanghai Pudong New Area, Shanghai, China; ^3^ Department of Cardiology, Renji Hospital Ningbo Branch, Shanghai Jiao Tong University School of Medicine, Ningbo, China

**Keywords:** cardiovascular-kidney-metabolic index, in-hospital mortality, intensive care unit, MIMIC-IV database, retrospective cohort study

## Abstract

**Background:**

The cardiovascular-kidney-metabolic index (CKMI), a novel functional indicator proposed in this study, aims to accurately reflect the functional status of the heart, kidneys, and metabolism. However, its ability to predict mortality risk in critically ill patients during their stay in the intensive care unit (ICU) remains uncertain. Therefore, this study aims to validate the correlation between the CKMI during hospitalization and all-cause mortality.

**Methods:**

The study utilized the Medical Information Mart for Intensive Care IV 2.2 (MIMIC-IV) dataset for a retrospective analysis of cohorts. The cohorts were divided into quartiles based on CKMI index levels. The primary endpoint was all-cause mortality during ICU and hospital stay, while secondary endpoints included the duration of ICU stay and overall hospitalization period. We established Cox proportional hazards models and employed multivariable Cox regression analysis and restricted cubic spline (RCS) regression analysis to explore the relationship between CKMI index and all-cause mortality during hospitalization in critically ill patients. Additionally, subgroup analyses were conducted based on different subgroups.

**Results:**

The study enrolled 1,576 patients (male 60.79%). In-patient and ICU mortality was 11.55% and 6.73%. Multivariate COX regression analysis demonstrated a significant negative correlation between CKMI index and the risk of hospital death [HR, 0.26 (95% CI 0.07–0.93), *P* = 0.038] and ICU mortality [HR, 0.13 (95% CI 0.03–0.67), *P* = 0.014].RCS regression model revealed that in-hospital mortality (*P*-value =0.015, P-Nonlinear =0.459) and ICU mortality (*P*-value =0.029, P-Nonlinear =0.432) increased linearly with increasing CKMI index. Subgroup analysis confirmed consistent effect size and direction across different subgroups, ensuring stable results.

**Conclusion:**

Our research findings suggest that a higher CKMI index is associated with a significant reduction in both in-hospital and ICU mortality among critically ill patients. Therefore, CKMI index emerges as a highly valuable prognostic indicator for predicting the risk of in-hospital death in this population. However, to strengthen the validity of these results, further validation through larger-scale prospective studies is imperative.

## Introduction

The intricate interplay and significant impact of cardiovascular, renal, and metabolic functions on patient outcomes make them pivotal in critically ill individuals (1–3). Throughout the entire duration of Intensive Care Unit (ICU) stay, a comprehensive evaluation of various biomarkers and indices is regularly conducted to ascertain prognosis and guide treatment decisions. Conventional markers such as left ventricular ejection fraction (LVEF), estimated glomerular filtration rate (eGFR), and triglyceride-glucose index (TyG) have been linked to adverse outcomes in critically ill patients (4–6).

LVEF is a critical indicator of cardiac function, reflecting the proportion of blood expelled by the left ventricle with each heartbeat. As an established marker of cardiovascular health, it has demonstrated associations with mortality in various patient populations (7). The eGFR is considered to be a more precise indicator of renal function than the creatinine level alone. In patients with chronic kidney disease, a decreased eGFR is linked to adverse cardiovascular outcomes and increased mortality (8). The TyG, which represents the degree of insulin resistance, has been associated with elevated cardiovascular risk and increased mortality in individuals diagnosed with metabolic syndrome (9, 10).

In our study, we aimed to integrate three key indicators, namely LVEF, eGFR, and TyG, into a novel comprehensive index known as the Cardiovascular- Kidney- Metabolic index (CKMI). This comprehensive index is specifically designed for a thorough evaluation of cardiovascular, kidney, and metabolic functions in critically ill patients, as well as assessing the prognostic value of CKMI in predicting overall mortality during ICU hospitalization. To the best of our knowledge, this pioneering research represents the first attempt to amalgamate these three indicators into a unified index and evaluate its prognostic utility in critically ill patients. The CKMI is not simply a simplistic scoring system for physiological indicators, but rather an all-encompassing and systematic assessment tool for evaluating physiological stress and multi-organ function. It integrates crucial health indicators from the cardiovascular, renal, and metabolic systems. This interdisciplinary approach surpasses conventional scoring systems like APACHE II and SOFA (11, 12), which typically focus solely on acute physiological changes and organ failure while neglecting to fully consider the significant impact of cardiac, renal, and metabolic systems on patients’ overall physiological state. By comprehensively considering multiple key physiological parameters, the CKMI can accurately identify high-risk patients and provide robust support for clinical decision-making. In comparison to traditional scoring systems, the CKMI exhibits substantial improvements in predictive accuracy and clinical applicability, thereby offering a reliable scientific foundation for patient treatment and prognosis management.

The primary objective of this retrospective cohort study is to investigate the association between serum creatine kinase levels during hospitalization and overall mortality in critically ill patients. Data analysis was conducted using the Medical Information Mart for Intensive Care IV 2.2 (MIMIC-IV) database. By utilizing this innovative biomarker to identify high-risk patients, clinicians may be able to personalize treatment strategies more effectively and enhance the prognosis of critically ill individuals.

## Methods

### Data source

The present study is a retrospective observational investigation, utilizing data obtained from the online international database MIMC-IV (version 2.2) (https://mimic.mit.edu). MIMC-IV represents a longitudinal single-center repository established by the Computational Physiology Laboratory at Massachusetts Institute of Technology(MIT), Beth Israel Deaconess Medical Center at Harvard Medical School(BIDMC), and Philips Medical (13). This comprehensive database encompasses information pertaining to patients admitted to BIDMC between 2008 and 2019.This dataset has undergone examination and certification to grant author (X.Q.) access (Record ID 62252237), and it is responsible for data extraction. The project has received approval from the Institutional Review Board of MIT and BIDMC. As patient health information remains anonymous in the database, individual consent is not required.

### Population selection

**Figure 1:**
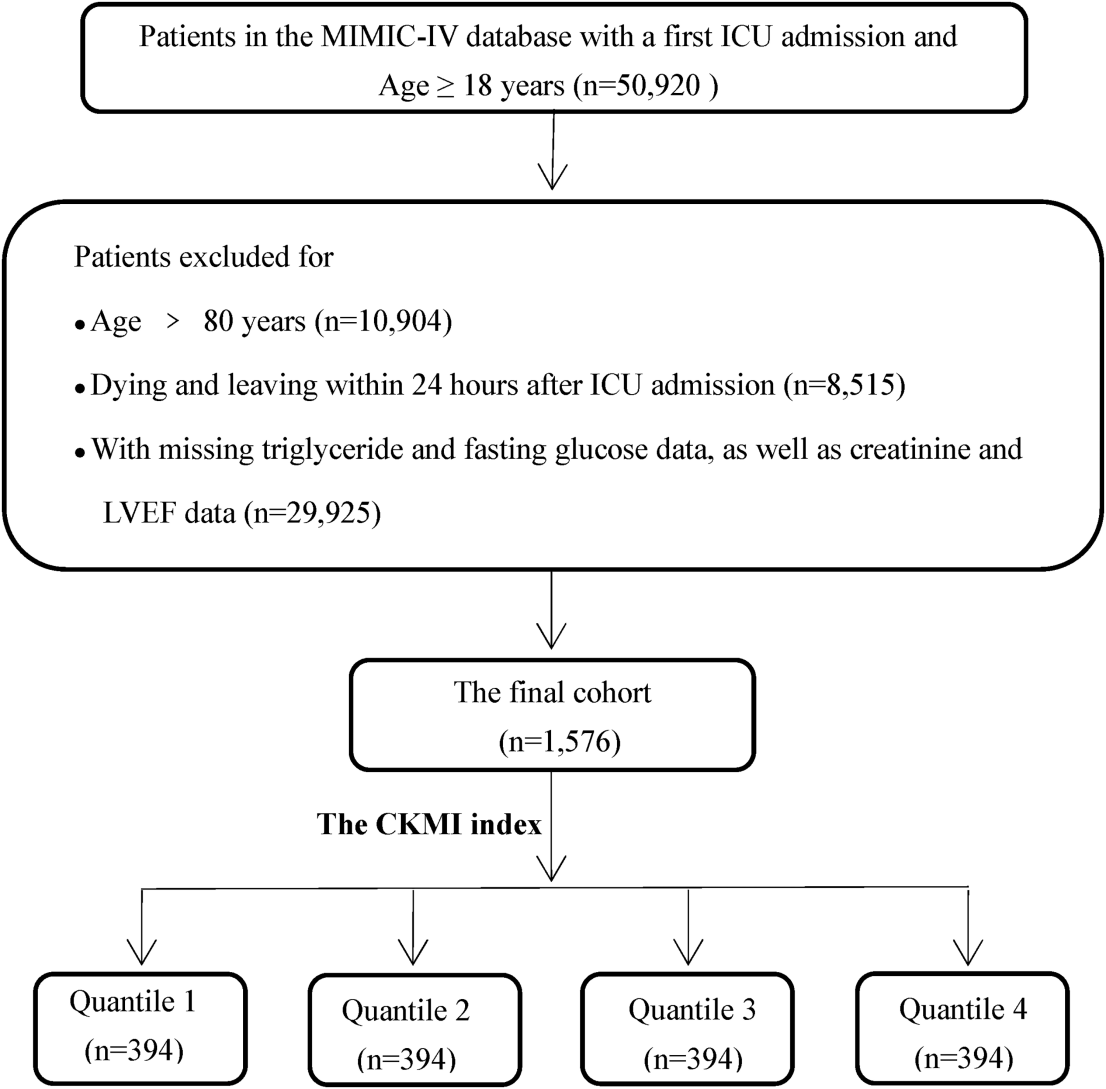
Flow chart of study participants.

The inclusion criteria for this study were as follows: (1) 18 years aged 80 years; (2) admission to the ICU; (3) availability of CKMI index calculation for patients; (4) ICU stay exceeding 24 h. In total, 1,576 patients were enrolled in the study and divided into four groups based on the CKMI index quartile. Please refer to Figure 1 for a detailed explanation of the research methodology.

### Data extraction

The baseline patient characteristics were obtained utilizing Structured Query Language (SQL) along with PostgreSQL (version 14.2). These attributes included patient demographic details comprising of age, gender, body mass index (BMI) as well as ethnicity. Additionally, vital signs like heart rate (HR), systolic blood pressure (SBP), diastolic blood pressure (DBP), mean arterial pressure (MAP), arterial oxygen saturation (SpO2), and body temperature (T) were recorded. The severity upon admission was evaluated based on the Sequential Organ Failure Assessment (SOFA) score, Acute Physiological Score III (APS III), systemic Inflammatory response syndrome (SIRS) score, Simplified Acute Physiological Score II (SAPSII), Oxford Acute Disease Severity Score (OASIS score), and Glasgow Coma Scale (GCS score). Intravenous vasoactive agents, including dobutamine, dopamine, and norepinephrine, are utilized. Laboratory test results encompass red blood cell (RBC), white blood cell (WBC), platelet, hemoglobin level, albumin concentration, serum creatinine (Scr) level as well as sodium, potassium and calcium ion concentrations. Additionally included are fasting blood glucose (FBG) value; glycated hemoglobin (HbA1c) level; anion gap; triglyceride (TG); total cholesterol (TC); high-density lipoprotein cholesterol (HDL-C); low-density lipoprotein cholesterol (LDL-C); alanine aminotransferase (ALT), and aspartate aminotransferase (AST). Cardiac ultrasonography findings represent the mean values of left ventricular ejection fraction (LVEF) during intensive care unit stay.Additionally, the following comorbidities were extracted from the MIMIC-IV database: coronary heart disease (CHD), congestive heart failure (CHF), myocardial infarction (MI), hypertension, diabetes, hyperlipidemia, chronic kidney disease (CKD), acute kidney injury (AKI), chronic obstructive pulmonary disease (COPD), respiratory failure (RF), stroke, liver disease (LD), pneumonia, sepsis and cancer.

The CKMI index is calculated using the following formula:

CKMI index = In [LVEF(%) × eGFR(ml/min/1.732)/2]/TyG Index.

The TyG index was calculated by employing the following formula, which takes into account levels of TG and FBG (14):

TyG Index = ln [TG (mg/dl) × FBG (mg/dl)/2]

Notably, the CKD-EPl equation for estimating GFR, developed in 2021 (in ml/min/1.73 m^2^), does not incorporate a race coefficient (15): Female and SCr ≤0.7 mg/dl: 143 × (SCr/0.7)^−0.241^ × 0.9938^age in years^ Female and SCr >0.7 mg/dl: 143 × (SCr/0.7)^−1.200^ × 0.9938^age in years^ Male and SCr ≤0.9 mg/dl: 142 × (SCr/0.9)^−0.302^ × 0.9938^age in years^ Male and SCr >0.9 mg/dl: 142 × (SCr/0.9)^−1.200^ × 0.9938^age in years^

### Primary outcomes and secondary outcomes

The primary outcomes measure of this study was the occurrence of all-cause mortality during hospitalization, encompassing both the ICU and general ward settings. Secondary outcomes included the duration of ICU stay and overall hospitalization period.

### Statistical analysis

To provide a comprehensive and easily understandable representation of data distribution, we conducted an extensive review of relevant literature and categorized CKMI into four groups based on quartiles (16). Continuous variables were reported as mean ± standard deviation (SD) or median quartile range (IQR), while categorical variables were presented as total and frequency (%). Pairwise comparison of continuous variables was conducted using Student's *t*-test, and multi-group comparison was performed using one-way ANOVA. Chi-square test was applied for pairwise comparison of categorical variables. After screening, more than 10% of variables with missing values are excluded from the analysis. For variables with missing values less than 10%, we employ multiple interpolation techniques to process and impute the missing data using the most appropriate dataset. Additionally, for variables exhibiting outliers, we apply a screening method based on the 1st and 99th percentile cutoff points. The Kaplan-Meier (K-M) curve and Cox proportional risk model were employed to assess the association between the CKMI index and the risk of in-hospital mortality. Only those variables exhibiting a significance level of *p* < 0.05 among the CKMI quartile groups were included in the multivariate model, considering baseline variables. Furthermore, multicollinearity was assessed using the variance inflation factor (VIF) to ensure independence of selected variables, with a proposed VIF value of 5 adopted based on previous research experience (17). Based on clinical expertise and relevant literature, we meticulously selected covariates that are closely associated with the research outcomes and further identified statistically significant covariates through univariate Cox regression analysis. Subsequently, three Cox proportional hazards models were constructed using the aforementioned approach: Model A, which included no adjustments; Model B, adjusted for age and BMI; and Model C, which further incorporated comorbidities such as CHD, CHF, hypertension, diabetes, stroke, sepsis, along with laboratory parameters including WBC, RBC, hemoglobin, albumin, HbA1c, and ALT in addition to the adjustments made in Model B. The association between the CKMI index and in-hospital mortality across various subgroups was examined through subgroup analysis. Additionally, the dose-response relationship between the CKMI index and mortality was investigated using restricted cubic splines (RCSs). Finally, receiver operating characteristic (ROC) curve analysis was performed to evaluate predictive ability alongside sensitivity and specificity. In addition, the effectiveness and robustness of the prediction model were evaluated through stepwise regression analysis, cross-validation analysis, as well as assessment of parameter correlation and interaction.

## Results

**Figure 2:**
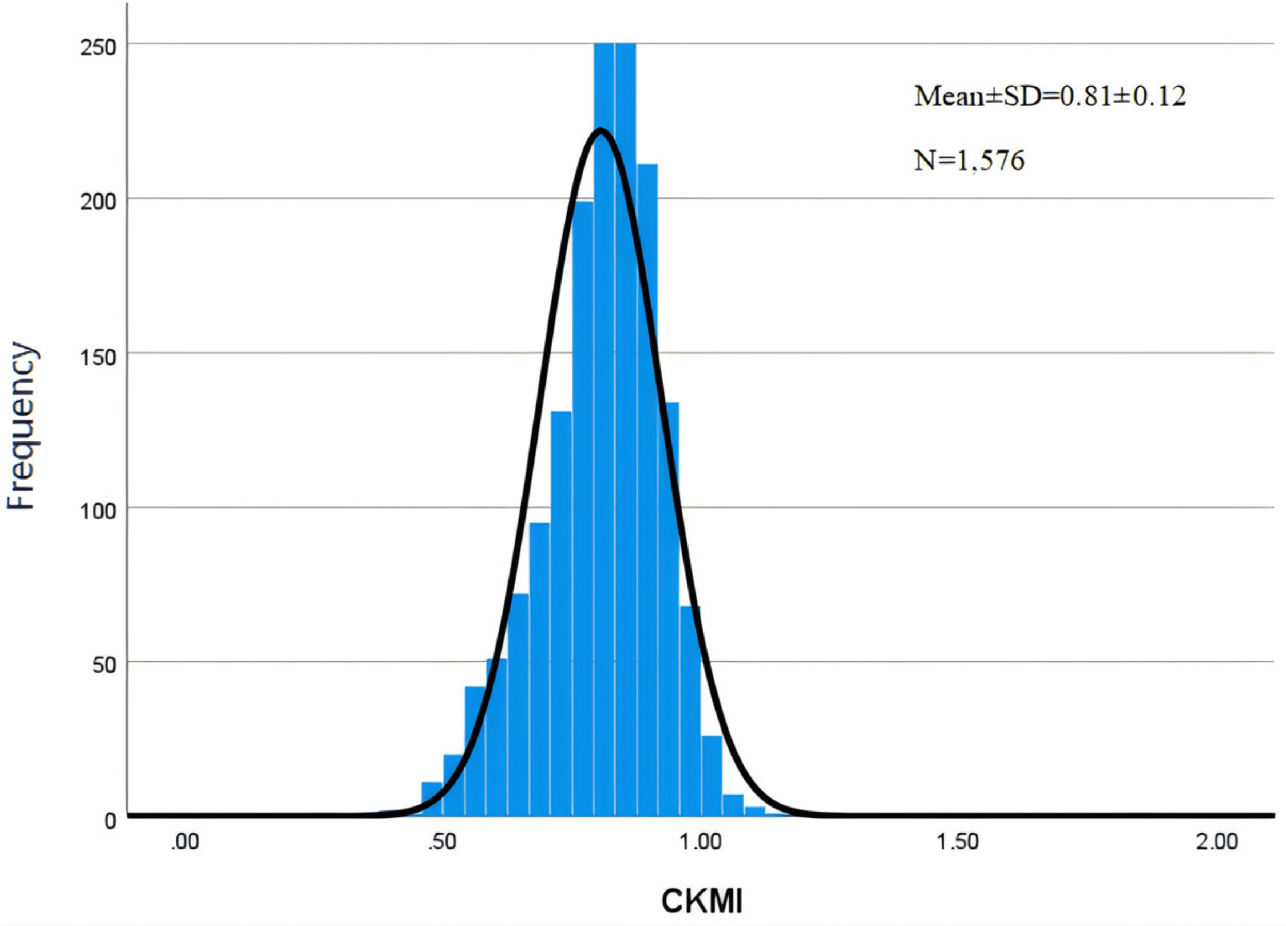
Histogram of CKMI.

A total of 1,576 patients were included in the study. The mean age of enrolled patients was 60.00 ± 13.45 years, with a majority being males (958; 60.79%). The mean CKMI index value for all participants was determined to be 0.81 ± 0.12 (Figure 2). In-hospital and ICU mortality rates were observed at rates of 11.55% and 6.73%, respectively.

### Baseline characteristics

The baseline characteristics of the patients enrolled in this study are presented in Table 1. Patients were stratified into quartiles based on their admission CKMI index(Q1: 0.29–0.74; Q2: 0.74–0.82; Q3: 0.82–0.89; Q4: 0.89–1.85), with mean CKMI levels for each group being 0.65 ± 0.07, 0.78 ± 0.02, 0.85 ± 0.02, and 0.94 ± 0.06, respectively. Compared to the high-value group, patients with a lower CKMI index generally exhibit advanced age and higher BMI. In terms of vital signs upon admission, they present with an elevated heart rate, decreased blood pressure, and reduced SpO2 levels. The severity of disease at admission is also heightened, accompanied by an increased incidence of complications such as CHD, CHF, MI, diabetes, CKD, AKI, RF, pneumonia, sepsis, etc. Furthermore, there are significant elevations in WBC, serum potassium, Serum creatinine concentration, and FBG level; HbA1c content and anion gap are also notably increased. TC levels and ALT/AST enzyme activities are all elevated. Mechanical ventilation demand and CRRT treatment requirement escalate while rescue drug application frequency rises accordingly. However, these patients demonstrate a decreasing trend in the incidence of hypertension and stroke accidents. Additionally noted trends include decreased RBC, platelet, hemoglobin as well as albumin content reduction. Simultaneously observed are lower serum sodium concentration and serum calcium level along with diminished TC levels including HDL-C and LDL-C, finally yet importantly worth mentioning is the decrease in eGFR and LVEF. Moreover, with an increase in the CKMI index, there is a gradual decrease observed in the duration of ICU stay (7 days vs. 4 days vs. 3 days vs. 3 days, *P* < 0.001), length of hospitalization (17 days vs. 12 days vs. 10 days vs. 9 days, *P* < 0.001), all-cause ICU mortality (12.7% vs. 7.1% vs. 3.6% vs. 3.6%, *P* < 0.001) and in-hospital mortality (19.5% vs. 11.7% vs. 8.6% vs. 6.3%, *p* < 0.001).

**Table 1:** Baseline characteristics of the critically ill patients stratifed by the CKMI index quartiles.

Characteristic	CKMI				*p*-value
	Q1 [0.29,0.74], *N* = 394	Q2 [0.74,0.82], *N* = 394	Q3 [0.82,0.89], *N* = 394	Q4 [0.89,1.85], *N* = 394	
Demographic					
Age, years, *n* (%)					<0.001
<60	142 (36.0%)	171 (43.4%)	171 (43.4%)	204 (51.8%)	
≥60	252 (64.0%)	223 (56.6%)	223 (56.6%)	190 (48.2%)	
Gender, *n* (%)					0.712
Female	157 (39.8%)	145 (36.8%)	160 (40.6%)	156 (39.6%)	
Male	237 (60.2%)	249 (63.2%)	234 (59.4%)	238 (60.4%)	
Ethnicity, *n* (%)					0.393
White	230 (58.4%)	256 (65.0%)	236 (59.9%)	259 (65.7%)	
Black	45 (11.4%)	30 (7.6%)	38 (9.6%)	28 (7.1%)	
Asian	8 (2.0%)	7 (1.8%)	10 (2.5%)	13 (3.3%)	
Hispanic/Latino	13 (3.3%)	15 (3.8%)	17 (4.3%)	12 (3.0%)	
Other	98 (24.9%)	86 (21.8%)	93 (23.6%)	82 (20.8%)	
Weight, kg, median [IQR]	91 (75, 107)	89 (74, 107)	81 (71, 95)	80 (67, 95)	<0.001
Height, cm, median [IQR]	170 (163, 178)	173 (163, 178)	171 (163, 176)	172 (164, 177)	0.431
BMI, kg/cm^2^, *n* (%)					<0.001
<28	134 (34.0%)	148 (37.6%)	196 (49.7%)	213 (54.1%)	
≥28	260 (66.0%)	246 (62.4%)	198 (50.3%)	181 (45.9%)	
Vital signs					
HR, bpm, *n* (%)					<0.001
<80	98 (24.9%)	111 (28.2%)	149 (37.8%)	183 (46.4%)	
80–100	140 (35.5%)	157 (39.8%)	143 (36.3%)	134 (34.0%)	
≥100	156 (39.6%)	126 (32.0%)	102 (25.9%)	77 (19.5%)	
SBP, mmHg, median [IQR]	121 (104, 140)	125 (110, 144)	130 (109, 147)	130 (112, 149)	<0.001
DBP, mmHg, median [IQR]	67 (55, 80)	72 (60, 86)	72 (62, 84)	73 (63, 84)	<0.001
MBP, mmHg, median [IQR]	79 (68, 93)	85 (73, 98)	86 (75, 100)	87 (75, 99)	<0.001
SpO_2_, %, median [IQR]	97.0 (94.0, 99.0)	97.0 (94.3, 100.0)	98.0 (95.0, 100.0)	98.0 (95.0, 100.0)	0.088
Severity scores					
SOFA score, median [IQR]	7.0 (4.0, 11.0)	4.0 (2.0, 8.0)	3.0 (1.0, 6.0)	2.0 (1.0, 5.0)	<0.001
APSIII score, median [IQR]	59 (44, 77)	43 (32, 59)	36 (27, 50)	33 (24, 45)	<0.001
SIRS score, median [IQR]	3.00 (2.00, 4.00)	3.00 (2.00, 3.00)	3.00 (2.00, 3.00)	2.00 (2.00, 3.00)	<0.001
SAPSII score, median [IQR]	43 (33, 54)	32 (25, 43)	29 (22, 38)	27 (20, 35)	<0.001
OASIS score, median [IQR]	35 (29, 43)	31 (26, 37)	30 (24, 37)	28 (23, 34)	<0.001
GCS score, median [IQR]	15.00 (14.00, 15.00)	15.00 (14.00, 15.00)	15.00 (14.00, 15.00)	15.00 (13.00, 15.00)	0.068
Comorbidities					
CHD, *n* (%)					0.026
No	342 (86.8%)	350 (88.8%)	361 (91.6%)	365 (92.6%)	
Yes	52 (13.2%)	44 (11.2%)	33 (8.4%)	29 (7.4%)	
CHF, *n* (%)					<0.001
No	215 (54.6%)	267 (67.8%)	285 (72.3%)	345 (87.6%)	
Yes	179 (45.4%)	127 (32.2%)	109 (27.7%)	49 (12.4%)	
MI, *n* (%)					<0.001
No	314 (79.7%)	312 (79.2%)	329 (83.5%)	352 (89.3%)	
Yes	80 (20.3%)	82 (20.8%)	65 (16.5%)	42 (10.7%)	
Hypertension, *n* (%)					<0.001
No	276 (70.1%)	207 (52.5%)	187 (47.5%)	211 (53.6%)	
Yes	118 (29.9%)	187 (47.5%)	207 (52.5%)	183 (46.4%)	
Diabetes, *n* (%)					<0.001
No	203 (51.5%)	256 (65.0%)	302 (76.6%)	352 (89.3%)	
Yes	191 (48.5%)	138 (35.0%)	92 (23.4%)	42 (10.7%)	
Hyperlipemia, *n* (%)					0.601
No	256 (65.0%)	253 (64.2%)	259 (65.7%)	270 (68.5%)	
Yes	138 (35.0%)	141 (35.8%)	135 (34.3%)	124 (31.5%)	
CKD, *n* (%)					<0.001
No	269 (68.3%)	341 (86.5%)	367 (93.1%)	384 (97.5%)	
Yes	125 (31.7%)	53 (13.5%)	27 (6.9%)	10 (2.5%)	
AKI, *n* (%)					<0.001
No	110 (27.9%)	206 (52.3%)	283 (71.8%)	339 (86.0%)	
Yes	284 (72.1%)	188 (47.7%)	111 (28.2%)	55 (14.0%)	
COPD, *n* (%)					0.235
No	363 (92.1%)	360 (91.4%)	373 (94.7%)	370 (93.9%)	
Yes	31 (7.9%)	34 (8.6%)	21 (5.3%)	24 (6.1%)	
RF, *n* (%)					<0.001
No	166 (42.1%)	224 (56.9%)	249 (63.2%)	284 (72.1%)	
Yes	228 (57.9%)	170 (43.1%)	145 (36.8%)	110 (27.9%)	
Stroke, *n* (%)					0.016
No	355 (90.1%)	354 (89.8%)	346 (87.8%)	329 (83.5%)	
Yes	39 (9.9%)	40 (10.2%)	48 (12.2%)	65 (16.5%)	
HD, *n* (%)					0.990
No	355 (90.1%)	357 (90.6%)	357 (90.6%)	355 (90.1%)	
Yes	39 (9.9%)	37 (9.4%)	37 (9.4%)	39 (9.9%)	
Pneumonia, *n* (%)					<0.001
No	225 (57.1%)	237 (60.2%)	255 (64.7%)	285 (72.3%)	
Yes	169 (42.9%)	157 (39.8%)	139 (35.3%)	109 (27.7%)	
Sepsis, *n* (%)					<0.001
No	249 (63.2%)	315 (79.9%)	322 (81.7%)	345 (87.6%)	
Yes	145 (36.8%)	79 (20.1%)	72 (18.3%)	49 (12.4%)	
Cancer, *n* (%)					0.458
No	361 (91.6%)	348 (88.3%)	352 (89.3%)	356 (90.4%)	
Yes	33 (8.4%)	46 (11.7%)	42 (10.7%)	38 (9.6%)	
Laboratory tests					
WBC, K/ul, median [IQR]	13 (9, 19)	12 (8, 16)	11 (8, 14)	10 (7, 13)	<0.001
RBC, m/ul, median [IQR]	3.56 (3.05, 4.21)	3.90 (3.30, 4.44)	4.04 (3.43, 4.50)	4.03 (3.48, 4.50)	<0.001
Platelet, K/ul, median [IQR]	198 (136, 261)	204 (154, 266)	209 (158, 265)	207 (159, 266)	0.067
Hemoglobin, g/dl, median [IQR]	10.50 (9.10, 12.70)	11.70 (9.80, 13.30)	12.00 (10.20, 13.60)	12.20 (10.50, 13.60)	<0.001
Albumin, g/dl, median [IQR]	3.00 (2.60, 3.50)	3.20 (2.70, 3.61)	3.36 (2.82, 3.78)	3.50 (3.00, 3.87)	<0.001
Sodium, mEq/L, median [IQR]	138.0 (134.3, 140.0)	138.0 (136.0, 141.0)	139.0 (136.0, 142.0)	139.0 (136.0, 141.0)	<0.001
Potassium, mEq/L, median [IQR]	4.40 (3.90, 4.90)	4.10 (3.70, 4.50)	4.00 (3.70, 4.30)	3.90 (3.60, 4.20)	<0.001
Calcium, mg/dl, median [IQR]	8.20 (7.60, 8.80)	8.30 (7.73, 8.90)	8.50 (7.90, 8.90)	8.50 (8.03, 9.00)	<0.001
Glucose, mg/dl, median [IQR]	172 (121, 244)	144 (116, 193)	121 (103, 147)	106 (93, 126)	<0.001
HbA1c,%,median [IQR]	6.50 (5.90, 7.63)	6.10 (5.79, 6.98)	5.86 (5.66, 6.20)	5.70 (5.50, 5.90)	<0.001
Aniongap, mEq/L, median [IQR]	18.0 (15.0, 21.0)	15.0 (13.0, 17.0)	14.0 (12.0, 16.0)	14.0 (12.0, 15.8)	<0.001
TG, mg/dl, median [IQR]	207 (131, 343)	162 (119, 230)	129 (100, 175)	84 (65, 109)	<0.001
TC, mg/dl, median [IQR]	128 (110, 158)	140 (119, 174)	146 (121, 186)	143 (123, 175)	<0.001
HDL-C, mg/dl, median [IQR]	31 (24, 38)	35 (29, 41)	37 (30, 44)	43 (35, 54)	<0.001
LDL-C, mg/dl, median [IQR]	67 (55, 84)	72 (59, 102)	77 (60, 112)	77 (60, 103)	<0.001
LT, IU/L, median [IQR]	43 (22, 102)	38 (21, 94)	34 (21, 72)	31 (18, 65)	<0.001
AST, IU/L, median [IQR]	70 (32, 186)	57 (28, 159)	47 (27, 111)	41 (24, 92)	<0.001
Creatinine, mg/dl, median [IQR]	2.00 (1.30, 3.60)	1.00 (0.80, 1.40)	0.90 (0.70, 1.10)	0.70 (0.60, 0.90)	<0.001
eGFR, ml/min/1.73^2^, *n* (%)					<0.001
Stage5 <15	282 (71.6%)	82 (20.8%)	27 (6.9%)	9 (2.3%)	
Stage4 15–30	94 (23.9%)	205 (52.0%)	209 (53.0%)	136 (34.5%)	
Stage3 30–60	18 (4.6%)	105 (26.6%)	157 (39.8%)	243 (61.7%)	
Stage2 60–90	0 (0.0%)	2 (0.5%)	1 (0.3%)	5 (1.3%)	
Stage1 ≥90	0 (0.0%)	0 (0.0%)	0 (0.0%)	1 (0.3%)	
LVEF, %, *n* (%)					<0.001
<50	158 (40.1%)	103 (26.1%)	71 (18.0%)	36 (9.1%)	
≥50	236 (59.9%)	291 (73.9%)	323 (82.0%)	358 (90.9%)	
Treatment measures					
Ventilation, *n* (%)					<0.001
No	63 (16.0%)	69 (17.5%)	88 (22.3%)	117 (29.7%)	
Yes	331 (84.0%)	325 (82.5%)	306 (77.7%)	277 (70.3%)	
CRRT, *n* (%)					<0.001
No	294 (74.6%)	365 (92.6%)	379 (96.2%)	388 (98.5%)	
Yes	100 (25.4%)	29 (7.4%)	15 (3.8%)	6 (1.5%)	
Dobutamine, *n* (%)					<0.001
No	355 (90.1%)	383 (97.2%)	387 (98.2%)	393 (99.7%)	
Yes	39 (9.9%)	11 (2.8%)	7 (1.8%)	1 (0.3%)	
Dopamine, *n* (%)					<0.001
No	358 (90.9%)	373 (94.7%)	378 (95.9%)	382 (97.0%)	
Yes	36 (9.1%)	21 (5.3%)	16 (4.1%)	12 (3.0%)	
Norepinephrine, *n* (%)					<0.001
No	200 (50.8%)	275 (69.8%)	290 (73.6%)	339 (86.0%)	
Yes	194 (49.2%)	119 (30.2%)	104 (26.4%)	55 (14.0%)	
Events					
LOS Hospital, days, median [IQR]	17 (8, 28)	12 (6, 26)	10 (4, 21)	9 (5, 20)	<0.001
LOS ICU, days, median [IQR]	7 (3, 13)	4 (2, 10)	3 (1, 9)	3 (2, 5)	<0.001
Hospital mortality, *n* (%)					<0.001
No	317 (80.5%)	348 (88.3%)	360 (91.4%)	369 (93.7%)	
Yes	77 (19.5%)	46 (11.7%)	34 (8.6%)	25 (6.3%)	
ICU mortality, *n* (%)					<0.001
No	344 (87.3%)	366 (92.9%)	380 (96.4%)	380 (96.4%)	
Yes	50 (12.7%)	28 (7.1%)	14 (3.6%)	14 (3.6%)	

CKMI, cardiovascular-kidney-metabolic index; BMI, body mass index; HR, heart rate; SBP, systolic blood pressure; DBP, diastolic blood pressure; MBP, mean blood pressure; SOFA, sequential organ failure assessment; APSIII, acute physiology score III; SIRS, systemic infammatory response syndrome; SAPSII, simplifed acute physiological score II; OASIS, oxford acute severity of illness score; GCS, Glasgow coma scale; CHD, Coronary Heart Disease; CHF, congestive heart failure; MI, myocardial infarction; CKD, chronic renal failure; AKI, acute kidney injury; COPD, chronic obstructive pulmonary disease; RF, respiratory failure; HD, hepatic disease; WBC, white blood cell; RBC, red blood cell; HbA1c, hemoglobin A1c; TG, triglyceride; TC, total cholesterol; HDL, high density lipoprotein; LDL, low density lipoprotein; ALT, alanine aminotransferase; AST, aspartic transaminase; eGFR, estimated glomerular filtration rate; CRRT, continuous renal replacement therapy; LOS, length of stay; ICU, Intensive Care Unit.

### Primary outcomes

In this study, we constructed three Cox proportional hazards models to investigate the association between the CKMI index and in-hospital mortality as well as ICU mortality. The results demonstrated significant negative correlations between the continuous CKMI index and both in-hospital mortality [Model A: HR, 0.25 [95% CI 0.08–0.77], *P* = 0.016; Model B: HR, 0.25[95% CI 0.08–0.76], *P* = 0.015; Model C: HR, 0.26 [95% CI 0.07–0.93], *P* = 0.038] and ICU mortality [Model A: HR, 0.14 [95% CI 0.03–0.59], *P* = 0.008; Model B: HR, 0.15 [95% CI 0.03–0.62], *P* = 0.009; Model C: HR, 0.13 [95% CI 0.03–0.67], *P* = 0.014] across all three models, unadjusted Model A, partially adjusted Model B, and fully adjusted Model C. Notably, in model C where adjustments were made for variables related to population characteristics and confounding factors, each one-standard-deviation increase in CKMI led to a remarkable 74% reduction in in-hospital mortality and 87% reduction in ICU mortality. When considering the CKMI index as a categorical variable, there was no significant association observed between the CKMI index and hospitalization or ICU mortality in Group Q2 compared to the lowest quartile (Group Q1) across all three Cox proportional risk models. However, a significant correlation was found in Groups Q3 and Q4, indicating that higher quartile arrays were associated with lower risks when compared to lower quartile arrays. In Model C, following comprehensive adjustment for potential confounders, CKMI index Q3 and Q4 exhibited a significantly decreased risk of hospital mortality compared to CIMI index Q1 [Q1 vs. Q3: HR, 0.71 [95% CI 0.45–0.91], *P* = 0.023; Q4: HR, 0.53 [95% CI 0.32–0.87], *P* = 0.012]. Furthermore, there was an inverse correlation between the increase in CKMI index value and the escalation of risk level. Cox proportional hazards analysis was employed to investigate the association between CKMI index and ICU mortality, yielding consistent findings [Q1 vs. Q3: HR, 0.42 [95% CI 0.22–0.79], *P* = 0.007; Q4: HR, 0.44 [95% CI 0.23–0.85], *P* = 0.014] (refer to Table 2).

**Table 2:** Cox proportional hazard ratios (HR) for all-cause mortality.

Variables	Q1		Q2		Q3		Q4		CKMI	
	HR (95% CI)	*p*-value	HR (95% CI)	*p*-value	HR (95% CI)	*p*-value	HR (95% CI)	*p*-value	HR (95% CI)	*p*-value
Hospital mortality										
Model A	Ref.	–	0.75 (0.52, 1.08)	0.127	0.71 (0.47, 0.95)	0.035	0.58 (0.37, 0.91)	0.017	0.25 (0.08,0.77)	0.016
Model B	Ref.	–	0.74 (0.51, 1.06)	0.102	0.67 (0.45, 0.97)	0.042	0.54 (0.34, 0.85)	0.008	0.25 (0.08,0.76)	0.015
Model C	Ref.	–	0.75 (0.51, 1.11)	0.150	0.71 (0.45, 0.91)	0.023	0.53 (0.32, 0.87)	0.012	0.26 (0.07,0.93)	0.038
ICU mortality										
Model A	Ref.	–	0.72 (0.45, 1.15)	0.166	0.46 (0.25, 0.83)	0.010	0.52 (0.29, 0.93)	0.029	0.14 (0.03,0.59)	0.008
Model B	Ref.	–	0.71 (0.45, 1.13)	0.151	0.44 (0.24, 0.81)	0.008	0.49 (0.27, 0.88)	0.018	0.15 (0.03,0.62)	0.009
Model C	Ref.	–	0.74 (0.45, 1.21)	0.234	0.42 (0.22, 0.79)	0.007	0.44 (0.23, 0.85)	0.014	0.13 (0.03,0.67)	0.014

HRs, hazard ratios; CI, confidence interval; CKMI, cardiovascular-kidney-metabolic index; ICU, intensive care unit; BMI, body mass index; CHD, coronary heart disease; CHF, congestive heart failure; WBC, white blood cell; RBC, red blood cell; HbA1c, hemoglobin A1c; ALT, alanine aminotransferase. Model A: unadjusted covariates. Model B: adjusted by age and BMI. Model C: adjusted by age, BMI, CHD, CHF, hypertension, diabetes, stroke, sepsis, WBC, RBC, hemoglobin, albumin, HbA1c, and ALT.

**Figure 3:**
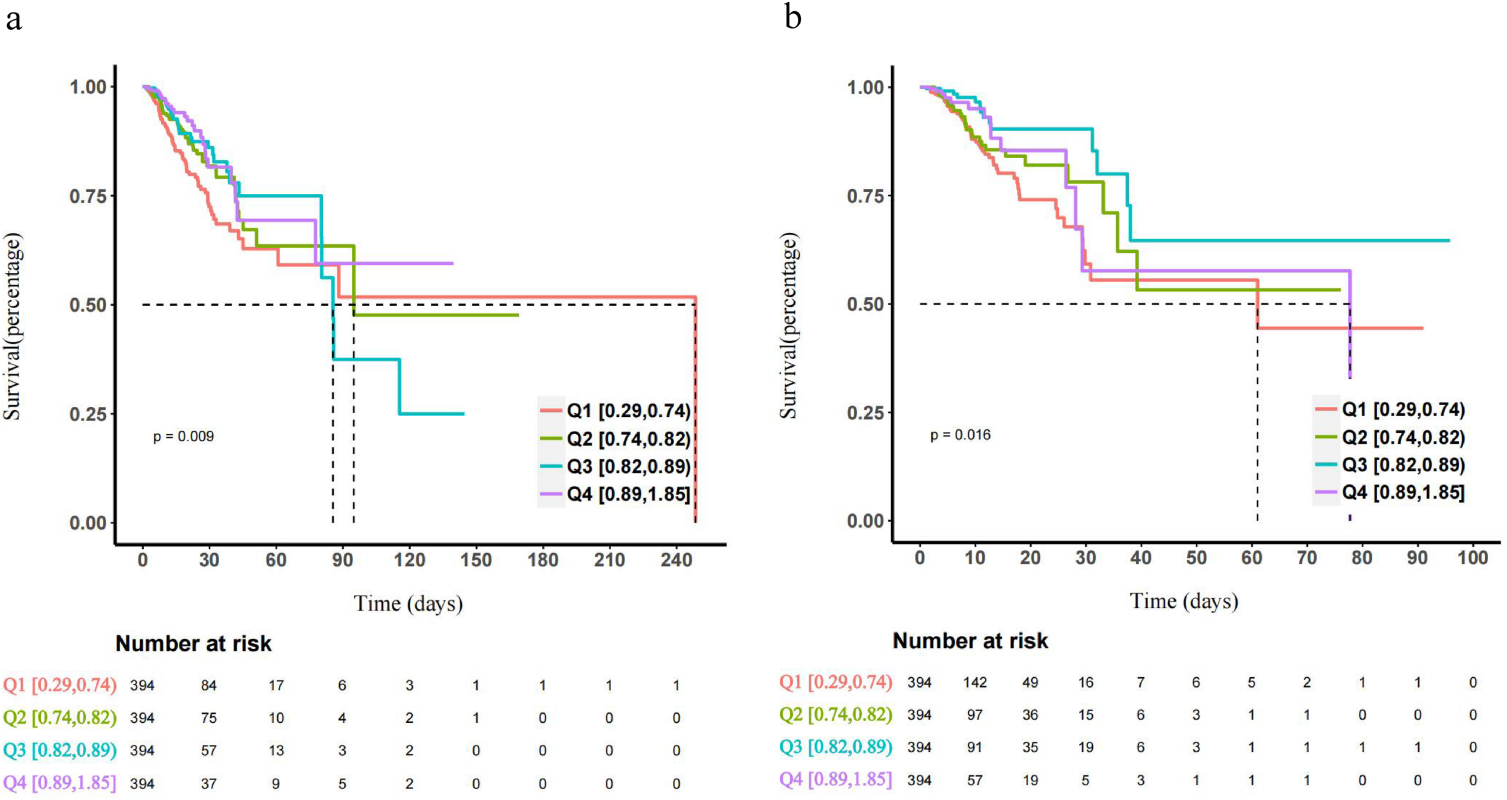
The Kaplan-Meier survival analysis curves depict the cumulative probability of all-cause mortality in quartile groups within the hospital **(a)**, and ICU **(b)**.

The incidence of major outcomes in each group, based on the CKMI index quartile, was analyzed using Kaplan-Meier survival analysis curve as depicted in Figure 3. Patients with a higher CKMI index exhibited a decreased risk of hospitalization and ICU mortality.

**Figure 4:**
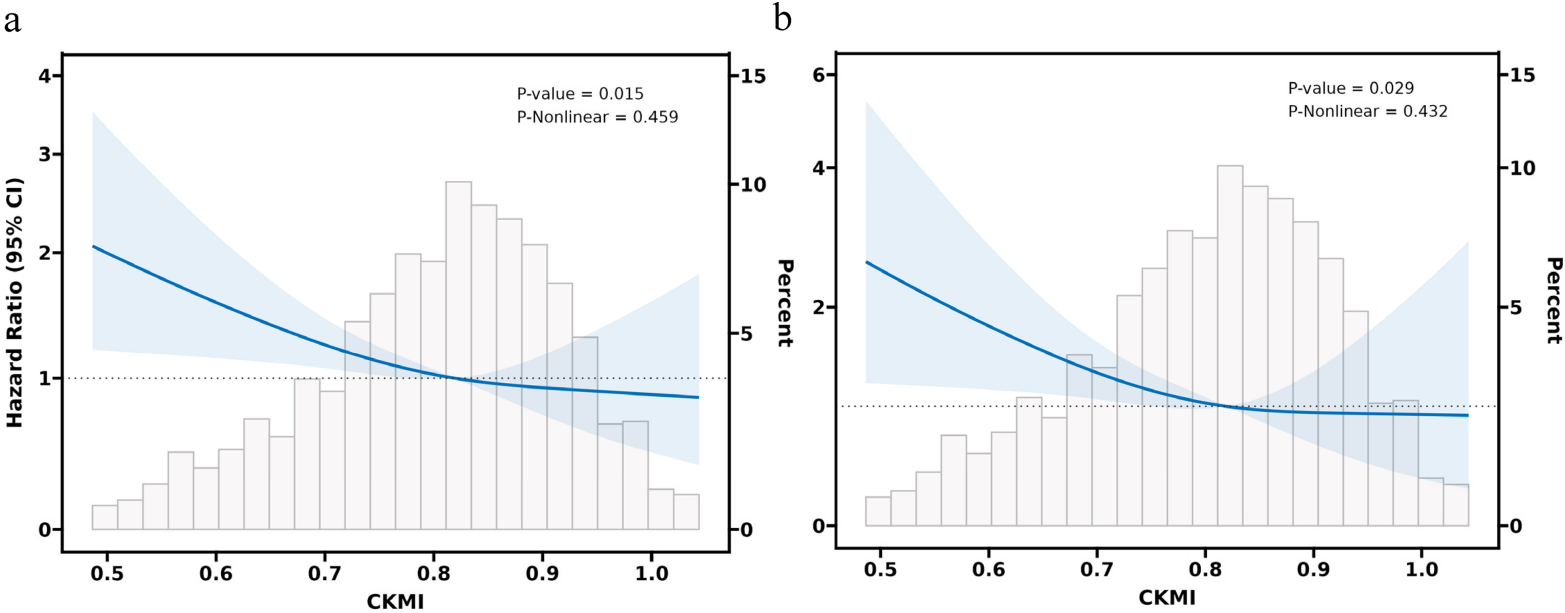
RCS analysis was conducted to examine the association between CKMI and all-cause mortality in both hospital and ICU settings. **(a)** The RCS curve illustrates the relationship between CKMI and all-cause hospital mortality. **(b)** The RCS curve demonstrates the correlation between CKMI and ICU mortality.

In the fully adjusted model C, a restricted cubic spline regression model was employed to demonstrate a consistent linear decline in both hospital mortality (*P*-value = 0.015, P-Nonlinear = 0.459) and ICU mortality (*P*-value = 0.029, P-Nonlinear = 0.432) as the CKMI index increased (Figure 4).

### ROC analysis of the CKMI index and its comparison with established severity scores

**Figure 5:**
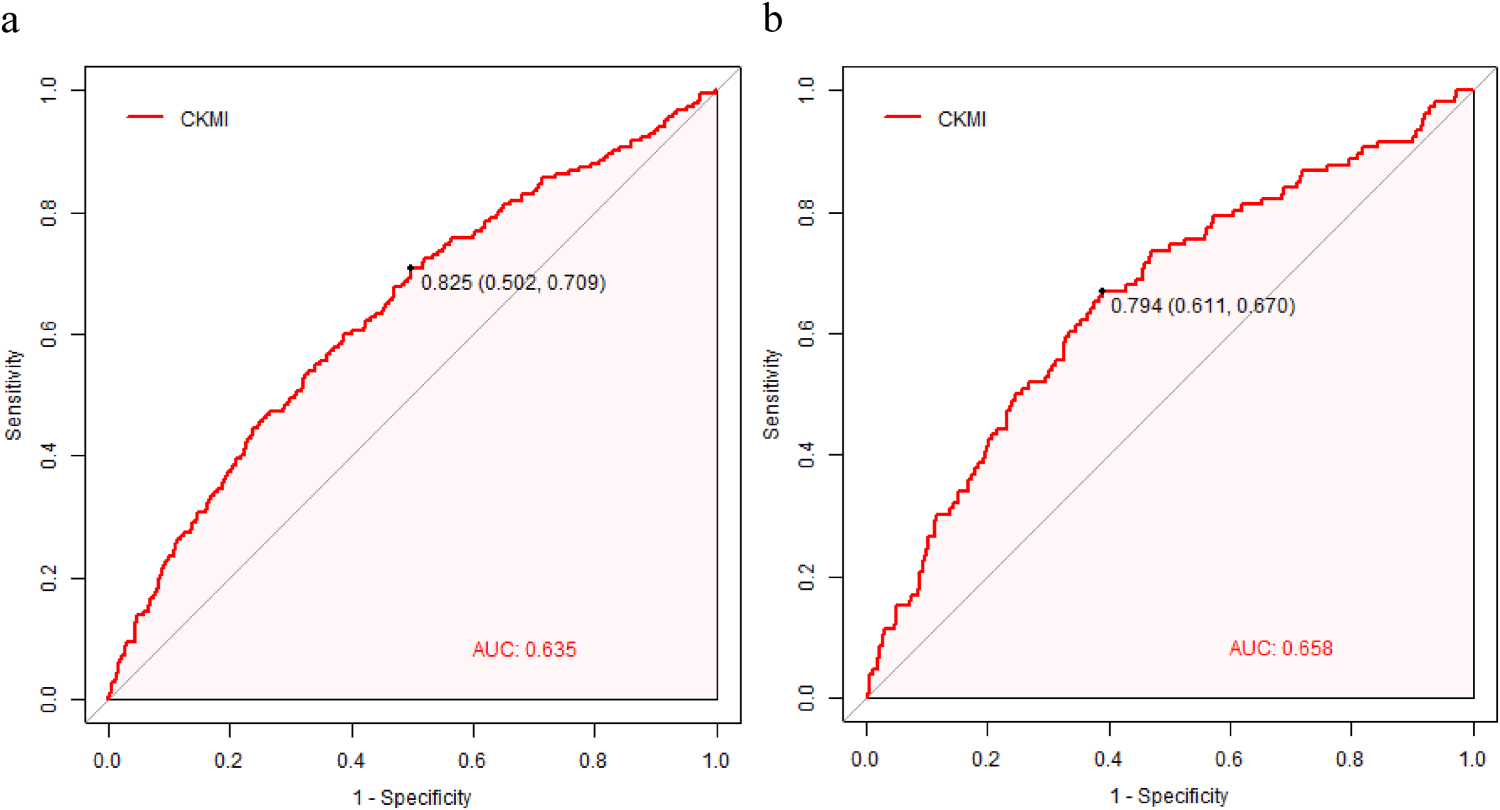
The CKMI correlation ROC curve for predicting in-hospital **(a)** and ICU mortality **(b)**.

The clinical efficacy of the CKMI index was evaluated using ROC analysis, revealing that the CKMI index exhibited a certain predictive value (AUC for in-hospital death: 0.635; AUC for ICU death: 0.658). The cutoff values for the CKMI index were determined as 0.825 and 0.794 for hospital deaths and ICU deaths respectively (Figure 5).

In order to conduct a more rigorous evaluation of the predictive performance of CKMI, we compared ROS analysis with established severity scoring tools such as SOFA, APSIII, and SIRS. In terms of predicting mortality in the ICU, CKMI exhibited a lower AUC value compared to established severity scores such as SOFA [0.759 (95% CI 0.716–0.802), *P* < 0.001], APSIII [0.796 (95% CI 0.753–0.839), *P* < 0.001], and SAPSII [0.775 (95% CI 0.732–0.818), *P* < 0001]. However, it demonstrated a significantly higher AUC value than OASIS [0.621 (95% CI 0.581–0.670), *P* = 0.043] and GCS 0.476 (95% CI 0.423–0.530, *P* < 0001)], while showing no statistical difference with SIRS [0.654 (95% CI 0.607–0.702), *p* = 0.923] (refer to Table 3).

**Table 3:** ROC analysis of the CKMI index and its comparison with established severity scores.

Predictor	ICU mortality AUC (95% CI)	The *p*-value compared to CKMI	Hospital mortality AUC (95% CI)	The *p*-value compared to CKMI
CKMI	0.658 (0.602–0.714)	–	0.635 (0.591–0.679)	–
SOFA	0.759 (0.716–0.802)	*P* < 0.001	0.728 (0.692–0.764)	*P* < 0.001
APSIII	0.796 (0.753–0.839)	*P* < 0.001	0.766 (0.732–0.801)	*P* < 0.001
SIRS	0.654 (0.607–0.702)	*P* = 0.923	0.624 (0.585–0.662)	*P* = 0.698
SAPSII	0.775 (0.732–0.818)	*P* < 0.001	0.774 (0.742–0.805)	*P* < 0.001
OASIS	0.621 (0.581–0.670)	*P* = 0.043	0.605 (0.545–0.659)	*P* = 0.039
GCS	0.476 (0.423–0.530)	*P* < 0.001	0.459 (0.418–0.500)	*P* < 0.001

In the prediction of hospital mortality rate, CKMI exhibited a lower AUC value compared to established severity scores such as SOFA [0.728 (95% CI 0.692–0.764), *P* < 0.001], APSIII [0.766 (95% CI 0.732–0.801), *P* < 0.001], and SAPSII [0.774 (95% CI 0.742–0.805), *P* < 0001]. However, it demonstrated a significantly higher AUC value than OASIS [0.605 (95% CI 0.545–0.659), *P* = 0.039] and GCS [0.459 (0.418–0.500), *P* < 0001], with no statistically significant difference observed when compared to SIRS [0.624 (95% CI 0.585–0662), *P* = 0.698] (refer to Table 3).

### Secondary outcomes

The results of multiple linear regression analysis revealed a significant negative correlation between the CKMI index and the length of stay in both ICU and general wards, even when not adjusting for confounding factors (LOS Hospital: β = −24.05, *P* < 0.001; LOS ICU: β = −14.51, *P* < 0.001) (refer to Table 4). This association remained consistent among hospitalized patients, even after partial (LOS Hospital: β = −25.99, *P* < 0.001; LOS ICU: β = −14.69, *P* < 0.001) or complete adjustment for confounders(LOS Hospital: β = −9.40, *P* = 0.031; LOS ICU: β = −7.83, *P* < 0.001) (refer to Table 4). These findings suggest that higher levels of CKMI may be indicative of longer hospital stays, thereby highlighting its potential as an effective indicator for assessing resource utilization in ICUs or hospitals, particularly in predicting critically ill patients who require extended periods of hospitalization.

**Table 4:** The correlation between the CKMI index and length of hospital stay (LOS).

Characteristic	β	95% CI	*p*-value
LOS Hospital			
Model A	−24.05	−32.01, −16.10	<0.001
Model B	−25.99	−34.05, −17.93	<0.001
Model C	−9.40	−17.95, −0.85	0.031
LOS ICU			
Model A	−14.51	−18.62, −10.40	<0.001
Model B	−14.69	−18.83, −10.56	<0.001
Model C	−7.83	−12.32, −3.34	<0.001

CI, confidence interval; CKMI, cardiovascular-kidney-metabolic index; ICU, intensive care unit; BMI, body mass index; CHD, coronary heart disease; CHF, congestive heart failure; WBC, white blood cell; RBC, red blood cell; HbA1c, hemoglobin A1c; ALT, alanine aminotransferase. Model A: unadjusted covariates. Model B: adjusted by age and BMI. Model C: adjusted by age, BMI, CHD, CHF, hypertension, diabetes, stroke, sepsis, WBC, RBC, hemoglobin, albumin, HbA1c, and ALT.

### Subgroup analysis

**Figure 6:**
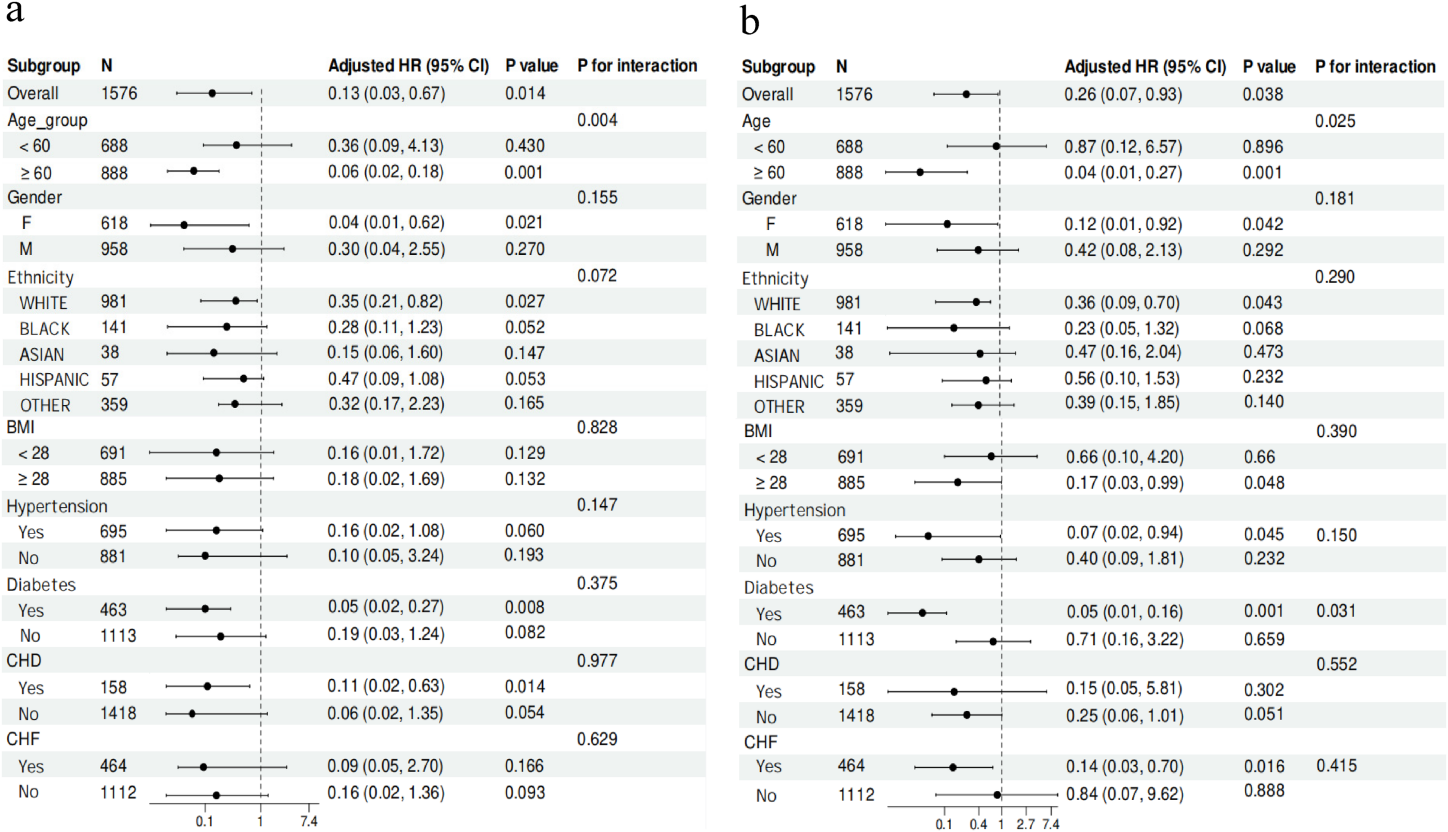
Subgroup analysis for the correlation between the CKMI index and the risk of ICU **(a)** and in-hospital **(b)** mortality in critically ill patients.

To further investigate potential disparities within the specific population, we conducted Cox regression analysis on various subgroups, encompassing crucial variables including age, gender, ethnicity, BMI ≥28, hypertension, diabetes, CHD, and CHF. By constructing subgroup forest plots, several noteworthy findings were revealed:

Upon further examination of the relationship between the CKMI index and ICU mortality, we observed a significant inverse association in specific subgroups including individuals aged ≥60 years [HR, 0.06 (95% CI 0.02–0.18), *P* = 0.001], females [HR, 0.04 (95% CI 0.01–0.62), *P* = 0.021], white ethnicity [HR, 0.35 (95% CI 0.21–0.82), *P* = 0.027], diabetic patients [HR, 0.05 (95% CI 0.02–0.27), *P* = 0.008], and those with CHD [HR, 0.11 (95% CI 0.02–0.63), *P* = 0.014]. In contrast, no such correlation was detected when comparing diabetic vs. non-diabetic patients, patients with BMI <28 vs. ≥28, hypertensive vs. non-hypertensive patients, and those with CHF compared to those without (Figure 6).

Among subgroups of individuals aged ≥60 years [HR, 0.04 (95% CI 0.01–0.27), *P* = 0.001], females [HR, 0.12 (95% CI 0.01–0.92), *P* = 0.042], white ethnicity [HR, 0.36 (95% CI 0.09–0.76), *P* = 0.043], those with a BMI ≥28 [HR, 0.17 (95% CI 0.03–0.99), *P* = 0.048], hypertensive patients [HR, 0.07 (95% CI 0.02–0.94), *P* = 0.045], diabetic patients [HR, 0.05 (95% CI 0.01–0.16), *P* = 0.001], and patients with CHF [HR, 0.14 (95% CI 0.03–0.70), *P* = 0.016], a significant inverse correlation was observed between the CKMI index and hospital mortality. However, it is important to note that no association between the CKMI index and hospital mortality was found when comparing patients with and without CHD (Figure 6).

Additionally, our study has revealed significant interactions between the CKMI index and other variables. Specifically, in terms of influencing hospital mortality, the CKMI index demonstrated noteworthy interactions with age (*P* for interaction = 0.025) and diabetes (*P* for interaction = 0.031). Similarly, when investigating factors impacting ICU mortality, a substantial interaction between the CKMI index and age (*P* for interaction = 0.004) was also observed (Figure 6).

In summary, this study provides robust evidence for comprehending the relationship between the CKMI index, diverse patient characteristics, and their clinical outcomes through meticulous subgroup analysis and exploration of interactions.

### The assessment of model value

We utilized stepwise regression analysis and cross-validation to assess the model, yielding a coefficient of determination (R-squared) of 0.9157, indicating that the model can account for 91.57% of the variability in the data. This outcome holds great significance, suggesting a robust fit and effective explanation of CKMI variations by the model. The mean squared error (MSE) was determined to be 0.0012, signifying minimal deviation between predicted values and actual values, thereby demonstrating high predictive accuracy. The stepwise regression analysis identified eGFR, TyG, and LVEF as pivotal variables within the model with statistically significant effects on CKMI (*p*-value < 0.05). Further cross-validation analysis revealed that among all tested models, the combination of three variables—LVEF + eGFR + TyG—achieved an exceptional R-squared score of 0.9038, indicating superior predictive performance compared to single parameter prediction models (LVEF, eGFR, TyG) or simple combination prediction models (LVEF + eGFR, LVEF + Tyg, eGFR + TyG). Moreover, it was observed that eGFR and TyG made substantial contributions towards predicting CKMI while LVEF played a relatively smaller role.

## Discussion

In this study, we introduced the CKMI as a novel functional indicator and validated its predictive validity for in-hospital and ICU all-cause mortality in critically ill patients using the extensive clinical database MIMIC-IV. CKMI, encompassing LVEF, eGFR, and metabolic index TyG, aims to comprehensively reflect the status of heart, kidney, and metabolic functions. The findings demonstrated a significant inverse correlation between CKMI and both in-hospital and ICU mortality, highlighting its potential as an important prognostic marker for predicting the risk of in-hospital mortality among critically ill patients.

As a crucial component of the CKMI index, LVEF serves as a pivotal indicator for assessing cardiac systolic function. Its decline typically heralds CHF or cardiac dysfunction, which are significant contributors to heightened in-hospital mortality (18). Numerous studies have demonstrated a strong association between reduced LVEF and the risk of cardiovascular events such as heart failure and myocardial infarction (7, 19). In critically ill patients, impaired cardiac function often results in inadequate circulating blood volume, subsequently compromising organ perfusion throughout the body and escalating the likelihood of death (20–22). Consequently, evaluating cardiac function through LVEF can indirectly reflect circulatory status and mortality risk among critically ill patients. A higher CKMI index signifies that individuals with superior cardiac function can endure greater physiological stress levels, thereby reducing hospitalization-related mortality.

As another crucial component of the CKMI index, eGFR is considered the gold standard for evaluating renal filtration function, and its decline often indicates renal function impairment (23). The kidney not only plays a pivotal role in waste excretion and fluid balance regulation but also actively participates in various physiological activities, including hormone secretion and blood pressure regulation (24–26). In critically ill patients, AKI is a common complication closely associated with mortality. AKI not only leads to the accumulation of metabolic waste and toxins in the body but also gives rise to significant issues such as electrolyte imbalances and acid-base disturbances, further exacerbating the patient's condition (27–29). Therefore, utilizing the CKMI index to assess renal function through eGFR can effectively predict the risk of death resulting from impaired renal function in critically ill patients.

The TyG index is a novel metabolic indicator that integrates levels of TG and FPG to assess insulin resistance and the risk of metabolic syndrome (30). In critically ill patients, metabolic dysfunction is a prevalent pathophysiological state closely associated with mechanisms such as inflammatory response and oxidative stress (31, 32). The TyG index serves as a valuable tool for evaluating metabolic status, with recent studies demonstrating its significant association with cardiovascular diseases (33), strokes (34), kidney diseases (35), and other pathological conditions (36–38). Moreover, emerging evidence suggests that the TyG index holds promise in predicting overall mortality and cardiovascular disease-specific mortality among the general population and critically ill patients (14, 39–41). A high CKMI index indicates a relatively favorable metabolic condition in patients, thereby reducing the likelihood of complications arising from metabolic abnormalities and consequently lowering in-hospital mortality.

The CKMI index is a comprehensive physiological health evaluation system that assesses cardiac function, renal function, and metabolic status in a holistic manner. In critically ill patients, these three aspects of functional status are intricately interconnected and mutually influential, collectively determining the prognosis of patients (4–6). CHF can result in circulatory disorders, which subsequently impact renal perfusion and metabolite clearance (42). Renal insufficiency may lead to toxin accumulation in the body, thereby increasing the burden on the heart and causing metabolic disturbances (43). Metabolic abnormalities can accelerate the progression of cardiovascular and renal diseases (44). Therefore, by simultaneously considering the key systems of the heart, kidney, and metabolism, the CKMI index achieves a comprehensive assessment of overall bodily function. In critically ill patients specifically, interdependencies among these three systems often exist and jointly determine patient outcomes. Consequently, utilizing the CKMI index enables a more comprehensive reflection of a patient's physiological state while enhancing prediction accuracy.

Currently, the prediction of in-hospital mortality for critically ill patients primarily relies on various models such as APACHE II score, SAPS II score, and SOFA score (45–47). However, these models predominantly rely on physiological parameters and medical history information for prediction. For instance, the APACHE II score focuses on assessing acute physiological status and chronic health conditions (45), while SOFA specifically evaluates sequential organ failure but often overlooks a comprehensive assessment of cardiac, renal, and metabolic states (47). CKMI offers a more comprehensive perspective by integrating indicators of cardiac, renal, and metabolic health to evaluate the physiological stress and multi-organ functional status of ICU patients. It may possess unique advantages in predicting ICU and in-hospital mortality rates. Metabolic status is a crucial indicator that reflects the body's energy metabolism and substance metabolism, which is closely associated with the development of various diseases. Previous studies have demonstrated that metabolic abnormalities, such as hyperglycemia and hypoalbuminemia, play a significant role in predicting adverse outcomes among ICU and hospitalized patients (48, 49). Therefore, incorporating metabolic status into scoring models aids in accurately assessing overall patient health and predicting unfavorable results. By comparing and analyzing different approaches, CKMI stands out for its uniqueness and innovation in integrating biological indicators like metabolic status. Traditional models often overlook these essential metabolic markers; however, they hold great significance when considering overall patient health and forecasting adverse outcomes. CKMI can more precisely reflect the comprehensive metabolic status of patients by including these metabolic indicators, thereby greatly contributing to clinical treatment guidance and patient prognosis assessment. In comparison to traditional scoring systems, CKMI provides a more comprehensive framework that assists clinicians in early identification of high-risk patients while developing personalized treatment plans. Moreover, the multidimensional comprehensive evaluation offered by CKMI may contribute to enhancing risk stratification and management strategies within complex ICU environments encompassing multiple variables.

Additionally, our study revealed a significant inverse correlation between CKMI levels and LOS in both the ICU and general wards, suggesting that patients with lower CKMI levels may necessitate prolonged hospitalization. The significant association between CKMI and LOS provides valuable insights into the utilization of ICU or hospital resources. Initially, patients exhibiting reduced levels of CKMI might require extended hospitalization and continuous monitoring, which could consequently increase the utilization of ICU or hospital resources. Consequently, by monitoring CKMI levels, we can promptly identify individuals who may require additional resources to ensure timely and effective treatment interventions. Secondly, the correlation between CKMI and LOS also implies that optimizing treatment strategies could potentially mitigate the adverse impact of CKMI on LOS, thus reducing patients’ length of stay in hospitals and minimizing resource utilization.

Finally, we observed overlapping Kaplan-Meier curves between Q2 and Q3, as well as between Q3 and Q4. After a thorough examination of the data and analysis process, we posit that the overlap of the curves may be attributed to several key factors: firstly, due to limited research resources in the database study, there might not be an adequate sample size to fully demonstrate significant differences in survival rates among different CKMI quartiles (Q1-Q4), despite efforts made to include a sufficient number of patients. The small sample size could result in less distinct differences between survival curves leading to overlapping phenomena. Secondly, heterogeneity exists within the patient population studied in terms of age, gender, underlying diseases, etc., which may cause variations in response to CKMI index among different patients and partially mask its predictive effect on survival rates for specific patient subgroups. Furthermore, the duration of follow-up can also impact the degree of separation between survival curves. If the follow-up time is insufficiently long enough, it may fail to capture significant differences in patient survival rates particularly at early stages. It should be emphasized that despite this phenomenon of overlap occurring; however,the CKMI index still retains certain predictive value especially for specific patient populations.

Moreover, in the subgroup analysis, a significant inverse association between CKMI and mortality risk was observed among female patients., while this correlation was not evident in male patients. Although the specific mechanisms are not fully understood, it is speculated that they may be attributed to several factors. Firstly, fluctuations in levels of sex hormones such as estrogen in women may exert profound effects on metabolic processes (50). estrogen exhibits anti-inflammatory, antioxidant, and cardiovascular protective effects which could potentially influence the relationship between CKMI and mortality risk among women (51). Secondly, females typically possess higher metabolism rates and distinct patterns of fat distribution which might contribute to more rapid elimination of metabolic waste and toxins from the body thereby alleviating metabolic stress; consequently impacting the association between CKMI and mortality risk (52). Additionally,females exhibit different biological characteristics and prognosis disparities compared to males when it comes to certain severe illnesses; these differences might result in a higher predictive value for CKMI among women (53, 54). In summary, an elevated CKMI during early stages reflects compensatory capacity of the body; whereas an elevation during later stages primarily indicates degree of organ failure. This variation could lead to gender-specific differences regarding predictive value of CKMI.Additionally, In order to investigate the disparities in metabolic capacity and predictive ability across different racial groups, we conducted a subgroup analysis based on the racial composition of the population. The findings revealed that CKMI exhibited a significant prognostic capability for mortality risk among Caucasians, while its efficacy was not observed in other ethnicities. Several potential factors may account for this discrepancy: firstly, substantial genetic and biological variations exist among diverse races, which could influence the association between CKMI and mortality risk (55); secondly, disparities in environmental factors and lifestyles might also impact the predictive performance of CKMI (56); additionally, critically ill patients from different racial backgrounds may exhibit distinct disease characteristics and patterns of complications, thereby affecting the applicability of CKMI as a prognostic indicator (57). It is important to note that due to relatively small sample sizes within other ethnic groups, statistical power is limited. Consequently, it is possible that statistically significant associations may go undetected even if they do indeed exist.

The CKMI index holds significant clinical application value, providing crucial prognostic information for patients in the early stages of ICU admission. For individuals with a low CKMI index, doctors can promptly implement intervention measures such as adjusting treatment plans and enhancing monitoring to mitigate the risk of mortality. Moreover, the CKMI index effectively reflects patient-specific differences and serves as a foundation for formulating personalized treatment strategies. Additionally, it allows dynamic adjustments based on changes in a patient's condition, offering an ongoing evaluation framework for physicians. Regular monitoring of the CKMI index enables timely modifications to treatment plans, ensuring optimal therapeutic outcomes for patients.

## Limitations

Despite the positive findings obtained in this study, several limitations should be acknowledged. Firstly, it is important to note that this study employed a retrospective analysis approach, which may introduce potential selection bias and information bias. Secondly, the accuracy of indicators such as LVEF, eGFR, and TyG upon which the calculation of CKMI index relies can be influenced by various factors. Moreover, it is worth mentioning that this study did not account for the impact of underlying diseases and treatment interventions on both CKMI index and in-hospital mortality.

Based on previous literature, we excluded patients aged ≥80 years due to potential physiological changes and differences in drug metabolism that may impact the interpretation of CKMI in elderly patients. Additionally, patients with ICU stays of less than 24 h were also excluded to mitigate the risk of missing data or inaccurate measurement of CKMI associated with shorter ICU stays. However, it is acknowledged that these exclusion criteria may limit the generalizability of our findings. Future studies are planned to investigate the performance of CKMI across different age groups and in patients experiencing rapid deterioration or early death within 24 h of ICU admission, aiming to enhance its applicability in diverse ICU patient populations.

Furthermore,we acknowledge the significance of dissecting the constituents of CKMI-related mortality rates to augment the efficacy of this study. This not only facilitates deeper insights but also fosters a comprehensive comprehension of the correlation between CKMI and mortality rates. Nevertheless, a major constraint of this study lies in our inability to procure specific cause-of-death information from the MIMIC database. This limitation curtails our capacity to conduct meticulous analyses on mortality rates associated with distinct components of CKMI (cardiovascular-related deaths, renal failure-related deaths, metabolic disease-related deaths). In prospective clinical studies, we intend to incorporate more participants who can furnish detailed records regarding causes of death so as to further investigate the relationship between CKMI and various causes of death.

To further validate the predictive efficacy of the CKMI index, future research should consider adopting a prospective design, increasing sample size, and incorporating additional influencing factors. Moreover, it is worth exploring the predictive value of the CKMI index in various disease types and age groups of patients, as well as investigating its potential synergistic effects when combined with other prediction models. Furthermore, studying the role of the CKMI index in informing treatment decision-making for critically ill patients can optimize clinical management plans.

## Conclusion

In summary, this study utilized the MIMIC-IV database to comprehensively investigate the efficacy of the CKMI index in predicting overall mortality during hospitalization for critically ill patients. The findings demonstrate a significant inverse association between the CKMI index and all-cause mortality within both hospitalization and ICU settings, suggesting its potential as a robust tool for prognosticating in-hospital death risk among critically ill patients. By integrating comprehensive assessments of cardiac function, renal function, and metabolic status, the CKMI index establishes a holistic physiological health evaluation system that offers clinicians more precise predictive evidence and personalized treatment guidance. However, further prospective studies with larger sample sizes are warranted to validate its predictive efficacy and clinical applicability.

## Data Availability

Publicly available datasets were analyzed in this study. This data can be found here: https://mimic.mit.edu/.
